# An integrated FoodNet in North East India: fostering one health approach to fortify public health

**DOI:** 10.1186/s12889-024-18007-w

**Published:** 2024-02-13

**Authors:** Madhuchhanda Das, Venencia Albert, Samaresh Das, Karma Gurmey Dolma, Tapan Majumdar, Pranjal Jyoti Baruah, Suranjana Chaliha Hazarika, Basumoti Apum, Thandavarayan Ramamurthy

**Affiliations:** 1 Indian Council of Medical Research (ICMR), Ansari Nagar New Delhi, 110029 India; 2 Center for Development of Advanced Computing, Kolkata, India; 3 Sikkim Manipal Institute of Medical Sciences, Sikkim, India; 4 Agartala Government Medical College, Tripura, India; 5 ICMR-Regional Medical Research Centre, Dibrugarh, Assam India; 6 Gauhati Medical College and Hospital, Assam, India; 7 Bankin Pertin General Hospital & Research Institute, Arunachal Pradesh, India; 8 ICMR-National Institute of Cholera and Enteric Diseases, Kolkata, India

**Keywords:** Bacteria, Diarrhea, Enteric infections, Foodborne diseases, Foodborne pathogen, FoodNet, Network evaluation, Northeast region, Outbreak investigation, Parasites, Surveillance network, Viruses

## Abstract

**Background:**

Food safety is a critical factor in promoting public health and nutrition, especially in developing countries like India, which experience several foodborne disease outbreaks, often with multidrug-resistant pathogens. Therefore, implementing regular surveillance of enteric pathogens in the human-animal-environment interface is necessary to reduce the disease burden in the country.

**Objective:**

To establish a network of laboratories for the identification of major food and waterborne pathogens prevailing in the northeast region of India through integrated surveillance of animal, food, human, and environment and investigate the antimicrobial susceptibility pattern of the pathogens of public health significance.

**Methods:**

The Indian Council of Medical Research (ICMR) has identified FoodNet laboratories; based on their geographical location, inclination to undertake the study, preparedness, proficiency, and adherence to quality assurance procedures, through an 8-step process to systematically expand to cover the Northeastern Region (NER) with comprehensive diagnostic capacities for foodborne pathogens and diarrhea outbreak investigations. Network initiated in the NER given the unique food habits of the ethnic population.

**Findings:**

This surveillance network for foodborne enteric pathogens was established in Assam, Arunachal Pradesh, Tripura, and Sikkim, and expanded to other four states, i.e., Manipur, Mizoram, Meghalaya, and Nagaland, thereby covering the entire NER by including nine medical and three veterinary centers. All these centers are strengthened with periodic training, technical support, funding, capacity building, quality assurance, monitoring, centralized digital data management, and website development.

**Results:**

The ICMR-FoodNet will generate NER-specific data with close to real-time reporting of foodborne disease and outbreaks, and facilitate the updating of food safety management protocols, policy reforms, and public health outbreak response. During 2020-2023, 13,981 food samples were tested and the detection of enteric pathogens ranged from 3 to 4%. In clinical samples, the detection rate of the pathogens was high in the diarrheal stools (8.9%) when 3,107 samples were tested. Thirteen outbreaks were investigated during the study period.

**Conclusion:**

Foodborne diseases and outbreaks are a neglected subject. Given the frequent outbreaks leading to the deaths of children, it is crucial to generate robust data through well-established surveillance networks so that a strong food safety policy can be developed for better public health.

## Introduction

Foodborne and waterborne diseases are emerging as an important public health challenge around the globe. Globalization of food trade, evolving agriculture and animal farming practices, and growing tourism inevitably allow the transmission of foodborne pathogens rapidly across distant borders [1, 2]. Unsafe food is estimated to cause 600 million cases of foodborne illnesses in humans and more than 400,000 deaths annually around the world [3]. A global burden of disease (GBD) study in 2015 estimated that foodborne diarrheal illness is the sixth leading cause of disability-adjusted life years (DALYs) [1]. The burden of foodborne diseases was maximum in Africa and South-East Asia with ≥9% of all DALYs. However, there is no systematic surveillance of foodborne diseases in most developing and underdeveloped countries. WHO recently published the global strategy for food safety 2022-2030 [4]. to reduce the burden of foodborne diseases. Additionally, the significance of food safety has been acknowledged as a critical factor for achieving the UN Sustainable Development Goals (SDGs), specifically, SDG 3 (Good health and well-being), SDG 6 (clean water and sanitation), and SDG 12 (responsible consumption and production) [5]. Center for Disease Control and Prevention (CDC) in the US has developed a foodborne disease active surveillance network (FoodNet), which has been collecting data on foodborne diseases since 1996 [6].

In India, foodborne diseases are the 5^th^ leading cause of disease burden and diarrhea is the third leading cause of childhood mortality [7]. Although, the country has a robust health infrastructure and disease burden estimation and reporting system through the Integrated Disease Surveillance Programme (IDSP), foodborne and waterborne diseases are not prioritized as a major public health problem. Despite the strength of public health initiatives, there is a notable absence of a dedicated emphasis on preventing and controlling diseases. Consequently, every year, foodborne outbreaks cause the deaths of hundreds of children. As it is a confined, self-limiting disease, data on the annual prevalence of foodborne diseases and outbreaks is scanty.

The northeast part of India is a special region of the country because of its geographic location, diverse population, and unique food practices. Despite of the immense potential and unexplored resources, this region remains an underdeveloped area due to its difficult terrain, limited infrastructural resources, political unrest, and underutilization of available health facilities. Foodborne diseases and outbreaks caused by microbial and environmental contaminants are perceived on a large scale in the Northeastern Region (NER), not only because of their inclination towards preserved, fermented, raw, boiled, and smoked foods but also the unique methods of preparation of different food items and a shortage of safe drinking water [8].

Due to the dearth of systematic data on foodborne diseases and outbreaks, the Indian Council of Medical Research (ICMR), New Delhi, has taken the initiative to establish a network of dedicated laboratories and conduct integrated surveillance of foodborne diseases in northeast India. This is the first foodborne disease surveillance network established in India.

Here, we present the process of developing the aforementioned network and the challenges faced while setting up the Sentinel Surveillance Network for Foodborne Pathogens (ICMR-FoodNet) in northeast India through eight systematic steps. Lessons learned during this process would help further expand the network in the other regions of India.

## Methodology

ICMR, an autonomous apex research organization under the Ministry of Health and Family Welfare (MoHFW), Government of India, is responsible for the formulation, coordination, and promotion of biomedical research. ICMR carries out national task force projects, which are goal-oriented, large-scale, multi-centric studies that specifically address significant public health issues to bridge the gap between research and policy.

### Study setting

Division of Epidemiology and Communicable Diseases, ICMR, which serves as the nodal coordinating center for planning, funding, and all logistic support for the ICMR-FoodNet.

### Identifying research gaps and formulating a Taskforce project

The risks emerging from the human-animal-environment interface, like food safety, and the threat of antimicrobial resistance (AMR) need to be addressed regularly. One Health (OH) research has recently gained importance through the G20 One Health initiative, which emphasizes the ‘*One Earth One Health concept’* to prevent future pandemics. This planning also maintains the ecosystem balance and also develops a viable strategy for achieving the UN Sustainable Development Goals (SDGs). Before the initiation of the project, an extensive literature review was performed, considering knowledge gaps and national research priorities for foodborne diseases. A concept proposal was developed and discussed in the ICMR Scientific Advisory Committee (SAC) Meeting in December 2018. With the recommendations of the SAC, this Taskforce project was planned to be centrally coordinated by ICMR, and implemented as a multi-centric study in the northeast region in a phased manner. The project covers three important aspects (1) Capacity building (2) Research (3) Public health implications.

The establishment of the ICMR FoodNet was achieved through eight systematic steps. The stepwise approach for the Multicenter Survey and Research Network is shown in Table 1.

**Table 1:** VIII step program for developing a multicenter survey and research network in India

STEP 1	Formulation of a standard protocol
STEP 2	Identify potential investigators and collaborating centers
STEP 3	Development of Standard Operating Procedures (SOP), Case Report Form (CRF), Guidelines, Memorandum of Agreement (MOA) and Ethical clearance
STEP 4	Identification of project sites and finalization of sample size
STEP 5	Performance monitoring and the evaluation of surveillance systems
STEP 6	Development of a FoodNet website and Web-based Data Repository, Mobile App, strain repository, retrieval and analytics platform
STEP 7	Strengthening the technical capacity and data management of the Centers
STEP 8	Expansion of the network for Integrated Foodborne Disease Surveillance in Northeast India

### STEP 1: Formulation of a standard protocol

The ICMR FoodNet initiative includes sentinel surveillance of enteric pathogens in food, humans, animals, and the environment. After the approval of SAC, a series of brainstorming sessions as well as review committee meetings were held to develop a unified standard protocol for the study. Initially, a protocol was developed for human and environmental study through the market, hospital surveillance, and outbreak investigations in four states, i.e., Assam, Sikkim, Tripura, and Arunachal Pradesh. Subsequently, the study was expanded to the other four states, i.e., Manipur, Mizoram, Meghalaya, and Nagaland. Animal husbandry surveillance has been included in the newly added states. Case definitions used in the project [e.g., “Diarrhea”, “Foodborne diseases”] followed the guidelines of the IDSP, Ministry of Health and Family Welfare, Government of India [9].

The primary objectives of the project are:

1. To identify the major circulating pathogens in humans, food items, animals, and the environment causing foodborne and waterborne diseases through the market, hospital, and animal husbandry surveillance and outbreak investigations.
2. Identification of sources and pathogens causing outbreaks of foodborne and waterborne diseases through systematic investigation of public health response.
3. Document genotyping and antimicrobial susceptibility patterns of identified bacterial pathogens.
4. Develop capacity for bacterial culture, antimicrobial susceptibility testing, molecular testing, metagenomics and outbreak investigations at the northeast institutes for foodborne and waterborne pathogens.

### STEP 2: Identify potential investigators and collaborating centers

Potential centers were identified through an invitation for expression of interest (EOI) and based on the availability of an equipped and functional bacteriology/microbiology laboratory, experienced microbiologist, and epidemiologists, and their networking with IDSP, Food Safety and Standards Authority of India (FSSAI) and state health authorities. A screening strategy involving two levels was implemented. A questionnaire consisting of 36 questions, broadly classified into three categories (1) institutional infrastructure (2) principal investigator (PI) expertise and manpower (3) microbiology laboratory and diagnostic facility was developed and distributed among the potential PIs. The questionnaire sought information on details such as the number of hospital beds, routine admission of diarrheal cases, publications on bacteriology/foodborne outbreak investigations, and willingness to join the network. Scoring was done by the experts based on the information provided by the PIs. Centers with higher scores were selected, and preference was also given to well established medical, veterinary and research institutes with adequate resources and experience in foodborne illness to capture diverse data from the NER.

**Fig. 1:**
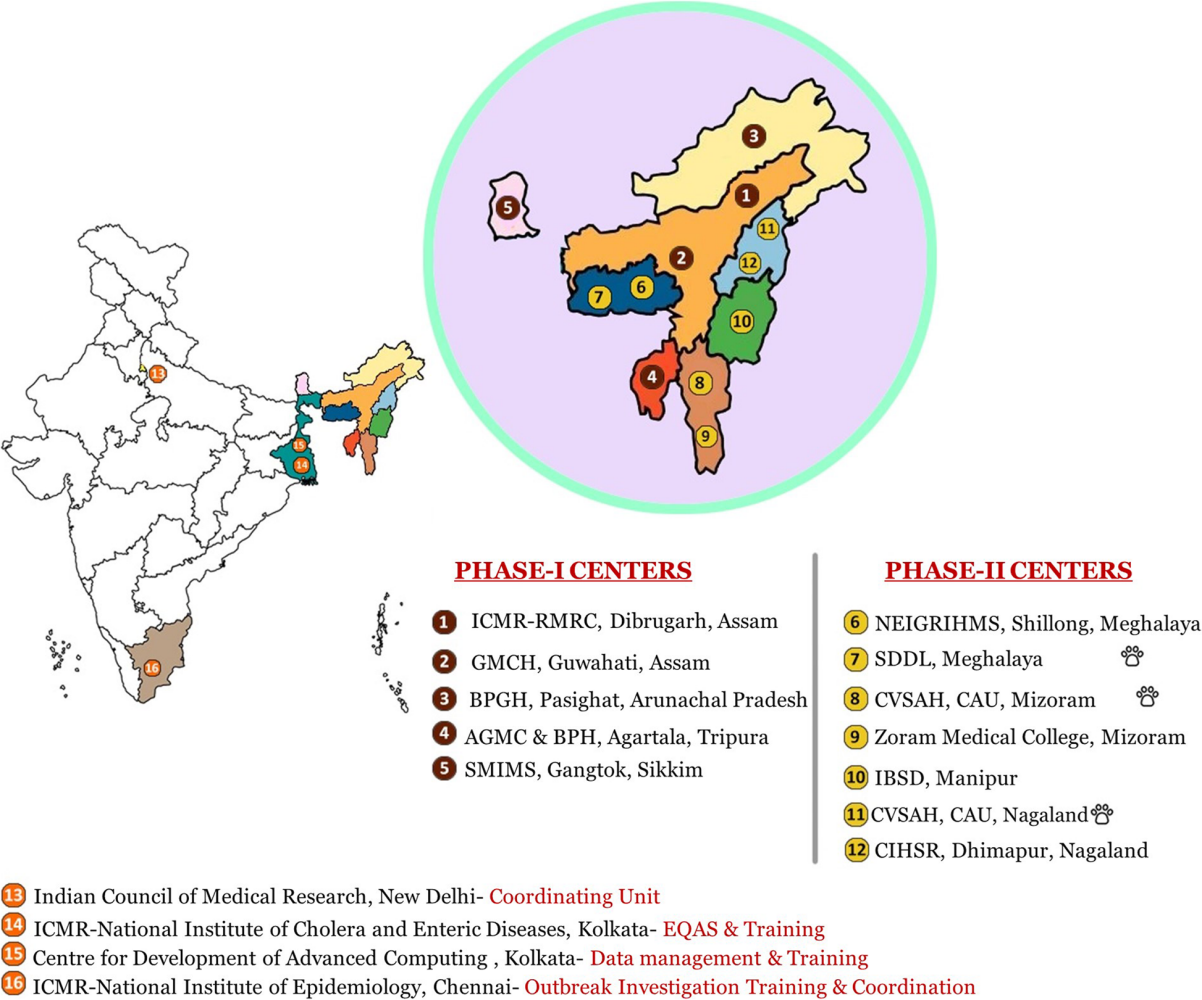
Geographical distribution of ICMR-FoodNet centres in Northeast India

The selected centers were invited to present their proficiency to conduct the study at the Technical Review Committee (TRC) meeting with subject experts. Based on the recommendation of the TRC and approval of the Director General (DG), ICMR, five centers were selected for funding in four states in Phase I. In Phase II, seven centers (three veterinary and four medical) were selected from the remaining four northeast states (Fig. 1). These centers were funded initially for three years and based on satisfactory evaluation and progress, and the project may be extended for two years as per ICMR task force policy.

Three more centers were added as partner institutes, i.e., Center for Development of Advanced Computing (C-DAC), Kolkata for centralized digital data management, ICMR-National Institute of Cholera and Enteric Diseases (NICED), Kolkata for External Quality Assurance Services (EQAS) and laboratory training and ICMR-National Institute of Epidemiology (NIE), Chennai for outbreak investigation training and monitoring (Fig. 1).

### STEP 3: Development of standard operating procedures (SOP), case report form (CRF), guidelines, memorandum of agreement (MOA) and ethical clearance

SOP for ICMR Foodborne Pathogen Survey and Research Network (North-East India) [10]. was developed for the isolation, identification and detailed characterization of foodborne pathogens from different samples and AMR testing. Guidelines and questionnaires for outbreak investigation, CRF as well as guidelines for primary patient care during diarrheal disease outbreaks, were also developed. A uniform data collection list of cooked, uncooked, preserved, refrigerated, and state-specific food items was also prepared [11]. MOA was developed and executed with the consultation of the ICMR legal cell to maintain work ethics between the FoodNet institutes and ICMR. Ethical clearance from the ICMR-Central Ethics Committee on Human Research (CECHR) was obtained (Reference Number: CECHR 003/2023). Additionally, approval from the ethical committees of each participating institute was obtained for the study.

### STEP 4: Identification of project sites and finalization of sample size

In each state, four districts covering the east, west, north, and south geographical zones were identified. Within each district, one market (market surveillance) was selected for collecting food samples, one hospital (hospital surveillance) was selected for collecting clinical samples and recording data on diarrheal disease, hepatitis (A & E), and typhoid cases, and one animal farm, poultry farm and slaughterhouse (animal husbandry surveillance) was selected for sampling (animal husbandry surveillance). Additionally, information on total hospital admissions was documented.

Foodborne and waterborne disease outbreak reported by the IDSP, or local news/media within the state was considered as per protocol to identify the possible source and causative pathogens. Sample sizes for food samples, stool samples, animal samples, and environmental samples to be collected per month per state were finalized.

### STEP 5: Performance monitoring and the evaluation of surveillance systems

Performance monitoring and assessment of the ICMR-FoodNet Centers involve comprehensive measures that include, (ii) Regular visits by the core committee members to assess laboratory setup, engage with stakeholders, and conduct field visits for each district, (ii) Monthly meetings with mentors, core committee members, and site PIs for swift issue resolution and regular data monitoring, (iii) Half-yearly reviews by the TRC to assess project progress reports, (iv) Centralized real-time data monitoring, enabling advanced outbreak detection and efficient data cleaning and analysis, (v) Periodic laboratory training, outbreak investigation training, and quality assurance (external and internal) programs for the generation of robust data, and (vi) Development of SOP, Outbreak investigation modules and questionnaire, CRF for uniform data collection and analysis across centers.

### STEP 6: Development of a foodnet website and web-based data repository, mobile app strain repository, retrieval and analytics platform

ICMR FoodNet website [11]. has been developed and deployed by C-DAC, Kolkata. This centralized, secured role-based web platform has been used by data collectors, lab technicians, and researchers for surveillance. Apart from the web-based application, a mobile App (ICMR-FoodNet App) was also developed for real-time data collection from hospitals, markets, animal farms and outbreak locations. Center-specific login credentials were provided for secure data entry. The main goal of this digital repository and analytical system highlights the occurrence of specific foodborne illnesses over time. By analyzing the collected data, the system identifies trends and fluctuations in the occurrence of illnesses. Additionally, it can attribute the infections caused by specific foods and food habits, thereby helping to understand the causes and sources of contamination.

**Fig. 2:**
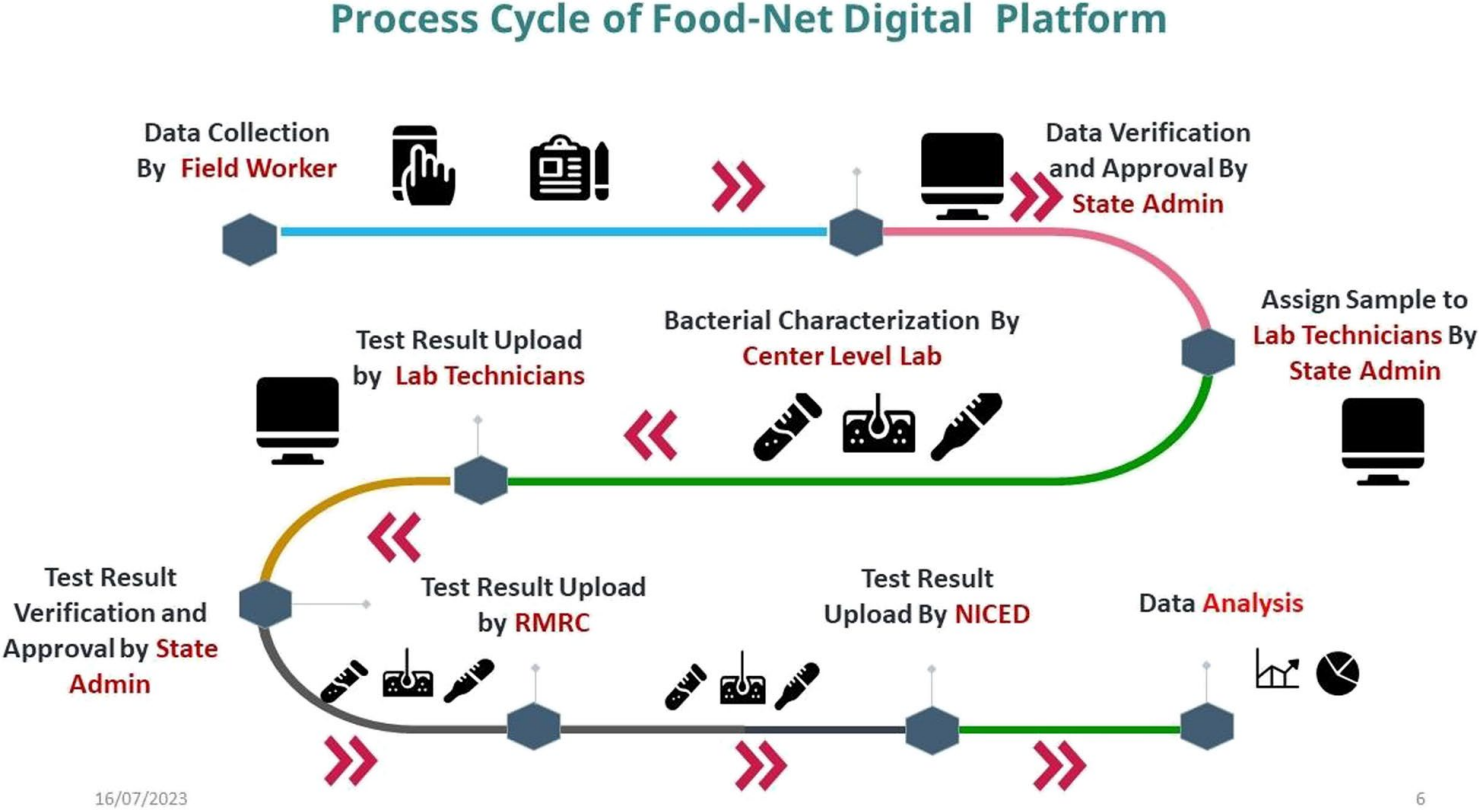
Process cycle of ICMR-FoodNet digital platform

Figure 2 illustrates the process of cleaning and analyzing the project-generated data. Each center employed a surveillance-specific CRF to collect information, which was subsequently input into a data repository on a secure cloud server. Following data submission, the center directed sample details to laboratory technicians for bacterial characterization, and the results were uploaded *via* the e-Lab Notebook Module. The identified bacterial strains were sent to ICMR-Regional Medical Research Centre (RMRC), Dibrugarh and ICMR-NICED, for pathogen confirmation.

Advanced data analytics, powered by artificial intelligence (AI) and machine learning (ML) techniques, were utilized to extract insights from the data repository. Analysis of long-term persistence of foodborne illness, identification of specific foods and dietary habits linked to these illnesses have been made through descriptive and statistical tools. A dashboard for collective information on the running status of project mandates, SOPs, and guidelines of the project was also designed and made available in the public domain. An online pathogenic strain repository is under development in ICMR-NICED, and RMRC.

### STEP 7: Strengthening the technical capacity and data management of the laboratory/centers

For uniform and reliable laboratory data generation, all research and technical staff recruited were trained to isolate and identify foodborne pathogens through conventional methods including serology and molecular techniques such as polymerase chain reaction (PCR) assays by ICMR-NICED, Kolkata. Internal quality assurance is being conducted by the RMRC, Dibrugarh. To generate additional quality data on new emerging enteric pathogens, an intense training program for metagenomics analysis was conducted for all centers at the Institute of Bioresources and Sustainable Development (IBSD) Sikkim. Digital data entry and data management training were also conducted during the commencement of the project.

The outbreak investigation part of this project has important public health implications, as reporting real-time data to the public and policymakers is crucial for formulating corrective interventions and implementing control strategies. NIE, Chennai as the coordinating center for outbreak investigation and training, has conducted a workshop for hands-on exercises in descriptive epidemiological analysis, analytical epidemiology, laboratory investigations, outbreak-based case studies, communication of outbreak recommendations, and common pitfalls during outbreak investigations. NIE provided training for clinicians, epidemiologists, and field staff of the project and also monitored outbreak investigations at the NER and assisted in the dissemination of the findings.

To provide technical guidance, an expert member from the TRC was identified as a mentor for each center. To assess the quality of laboratory work, external quality control strains were sent to each center for identification, serotyping, and antimicrobial susceptibility testing. A repository of all the identified bacterial strains is maintained at the ICMR-NICED, Kolkata and RMRC after detailed phenotypic and genetic characterization for future research.

One challenge faced was the initial disruption due to the nationwide COVID-19 outbreak and subsequent political unrest in the North East region and natural calamities that were hindering the work progress and monitoring in the first year.

This project has defined plan to deal with the problems that arise if the system at each stage has glitches in order to be able to fix them and keep the system running sustainably. Regular meetings with expert members play a crucial role in addressing micro-level issues. The program is seamlessly integrated with IDSP, FSSAI, and state health authorities, with their active involvement ensures the sustainability of the program. To systematically address problems, an eight-step plan was implemented through mentor-PI-Coordinator monthly meetings that include, (i) Define the problem, (ii) Find the root cause of the problem, (iii) Understand the severity of the problem, (iv) Brainstorm and evaluate possible solutions taking help from the ICMR and other collaborating centers, (v) Take containment action, (vi) Plan corrective action to fix the root cause, (vii) Implement the corrective action plan, and (viii) Follow up to ensure that the plan worked and not recurring.

### STEP 8: Expansion of the network for integrated foodborne disease surveillance in Northeast India

Successful establishment of the surveillance system in Phase I was followed by the initiation of Phase II in the remaining four northeast states, i.e. Manipur, Meghalaya, Mizoram, and Nagaland.

The animal husbandry surveillance carried out by the veterinary centers includes monitoring poultry farms, animal farms, and slaughterhouses, with the collection of samples from animals (such as feces and blood), animal handlers (including nail bed scrapings and hand wash samples), and the environment (such as water used for animal care and cleaning, soil, etc.). The incorporation of the OH surveillance program is aimed at the identification of the source (pathogen) attribution and transmission pathways. This comprehensive approach will contribute to a better understanding of the dynamics of foodborne pathogen transmission and antimicrobial resistance, facilitating the development of targeted interventions to mitigate health risks and ensure the safety of the food chain.

The screening of asymptomatic animals in the food supply chain enables the early identification and alerting of potential outbreaks, assisting in the prevention or reduction of disease transmission. Additionally, to facilitate targeted public health interventions for improved food safety, a qualitative situation analysis and behavioral components of food safety and hygiene practice among the food handlers will be taken up in the future. This is to improve understanding of local food and hygiene practices, and designing accurate and realistic intervention tools for food handlers.

## Results

ICMR has initiated the Phase I foodborne pathogens survey and ICMR-FoodNet in four NER states in October 2020. Initially, eleven centers were screened from all 8 states of the northeast. However, owing to the countrywide COVID-19 pandemic lockdown, 5 centers located in four states were selected based on their merit, namely (1) Agartala Govt. Medical College, Tripura, (2) Bankin Pertin General Hospital & Training Centre, Pasighat, Arunachal Pradesh, (3) Sikkim Manipal Institute of Medical Sciences, Gangtok, Sikkim, (4) Guwahati Medical College, Guwahati, Assam and (5) RMRC, Dibrugarh, Assam which is also served as the northeast coordinating center.

During the first year, laboratory upgradations were made in each center with the development of the facility to perform culture, antibiotic sensitivity testing, and molecular studies for foodborne pathogens. The diagnostic test portfolio was expanded to bacterial culture, microscopy, biochemical tests, serotyping, and PCR assays. Rapid diagnostic kits were provided to each center for identification of Rota, Adeno, and Norovirus. Molecular tests, genotyping, and antibiotic sensitivity patterns of identified bacterial pathogens were performed at the ICMR-NICED and RMRC. Metagenomic analysis of the selected samples (stool and suspected food samples) was performed at IBSD Imphal.

About 20 faculties and research scientists and technical staff were trained by the experts from NICED (laboratory techniques), C-DAC (Data collection and entry), NIE, (outbreak investigation and reporting) and IBSD (metagenomic analysis and anaerobic culture techniques). Control strains viz., *Vibrio cholerae, Shigella flexneri*, *Salmonella enterica* serovar Typhimurium*,* diarrheagenic *Escherichia coli* (ETEC, EPEC, EHEC, EAEC), *Listeria monocytogens*, *Bacillus cereus*, *Staphylococcus aureus*, *Yersinia enterocolitica, Campylobacter jejuni* and *Clostridioides* *difficile* were given to each center by the ICMR-NICED for correct identifications of the pathogens. The average EQAS score of 75% was achieved by each center.

From 2020 to 2023, 13,981 samples were tested in Phase I, including 4,362 cooked, 9,485 uncooked and 134 ethnic/traditional food items collected through the market surveillance. Overall sample positivity for enteric pathogens was 3.1% (432) [uncooked food items were 2.5% (236), cooked food items were 4.4% (192) and ethnic/traditional food items were 3.0 % (4)]. In addition, 3,297 clinical samples were tested, including 3,107 stools and 190 rectal swabs collected through the hospital survey. Sample positivity for enteric pathogens was 5.5% (172) and 8.9% (17) in stool and rectal swab samples, respectively. In total, 13 outbreaks occurred during the study period. From three outbreaks investigated in Sikkim, two in Tripura, and three in Assam, the causative pathogens were identified. The comprehensive investigation also included the profiling of AMR and the identification of the sources of infection. Outbreaks jointly investigated by the IDSP team through this project promptly contained the spread of outbreaks. Information regarding the source of infection was communicated to the state health authorities, enabling swift remedial actions.

A total of 196 enteric bacterial strains has been stored in the pathogen repository at NICED, [61 from humans, 118 from food items, 17 from the environmental samples] and 143 at RMRC, Dibrugarh [65 from humans, 74 from food items, 4 from the environmental samples)]. Antimicrobial susceptibility testing of *Salmonella enterica* serovar Typhi*, Shigella* spp*., Vibrio cholerae* non-O1, non-O139, *Bacillus cereus*, and *Staphylococcus aureus* were performed.

Based on the interim results of Phase I, in June 2023, Phase II was initiated in the remaining 4 northeastern states in seven sites, i.e., (1) Northeastern Indira Gandhi Regional Institute of Health, Shillong, Meghalaya (2) State Disease Diagnostic Laboratory, Animal Husbandry & Veterinary Department, Shillong, Meghalaya (3) IBSD, Imphal, Manipur (4) Zoram Medical College, Mizoram (5) College of Veterinary Sciences & Animal Husbandry, Central Agricultural University Aizwal, Mizoram (6) Christian Institute of Health Sciences and Research, Dimapur, Nagaland (7) College of Veterinary Sciences & Animal Husbandry Central Agricultural University, Peren, Nagaland out of the 13 screened centers.

Building upon the insights gained from Phases I and II and considering the availability of funds from ICMR/DHR, MoHFW, the next Phase will include the expansion of the network to four major metro cities in India, i.e., Delhi, Mumbai, Chennai, and Kolkata. The overarching plan is to systematically and progressively extend the network to encompass other states, with the ultimate goal of providing comprehensive coverage of the entire country.

### Challenges

Research in NER of India is challenging due to its geographical and infrastructural constraints, including difficult terrain, road conditions, heavy monsoon, land sliding, limited transportation, electricity shortage, lack of internet access in remote areas, and political unrest. The socio-cultural diversity and language barriers of the region requires effective communication. Furthermore, there is limited research infrastructure, opportunity and research funding, sparse networks, data collection, and quality control, especially in less developed regions. Additionally, The COVID-19 pandemic impacted foodborne disease surveillance through changes in food consumption patterns, food handling practices, and access to healthcare. Decline in the reporting of foodborne illness was a notable effect of the lockdown during 2020-2022 [12].

## Discussion

Foodborne diseases and outbreaks are neglected public health problems. As quality food and water are an integral part of healthy human life, there has been a growing emphasis on prioritizing research in the field of food safety in recent years. The government and public health organizations are working mutually to improve data collection, and analysis and to develop more effective interventions to prevent foodborne disease outbreaks. Early detection and response to foodborne disease outbreaks and effective control measures are critical to prevent mortality and morbidity. Also, the risks emerging from the human-animal-environment interface, like food safety risks, and the threat of AMR need to be addressed regularly. OH research has recently gained importance as Health Emergencies Prevention, Preparedness and Response (with a focus on OH & AMR) was identified as a priority Under India’s G20 Health Working Group. This underlines the need to promote the OH approach and create a framework for AMR stewardship and focus on enhancing primary health care, strengthening the health workforce, and improving essential health services and systems to address and combat various diseases, including waterborne illnesses [13].

In 1995, the United States successively established the Foodborne Diseases Active Surveillance Network for population-based sentinel surveillance to track trends for infections transmitted commonly through food [5]. This surveillance includes 15% of the US population (~50 million). FoodNet in the US accomplishes its work through active surveillance, surveys of laboratories, physicians, and the general population; and population-based epidemiologic studies collecting data on infections caused by the major pathogens such as *Campylobacter, Listeria*, *Salmonella*, *Shiga* toxin-producing *Escherichia coli*, *Shigella*, vibrios, and *Yersinia*. Other systems like PulseNet [14]. a nationwide network for molecular subtyping in the surveillance of foodborne diseases, CDC, initiative have also been successfully functional in identifying, investigating, tracing, and warning of foodborne disease outbreaks.

The European Food Safety Authority (EFSA) collects data on zoonoses, zoonotic agents, AMR, microbiological contaminants, and foodborne outbreaks across Europe [15]. Several other countries have foodborne disease surveillance programs to improve the safe food supply and prevent foodborne infections [6, 16–23]. including, Japan [16]. Iran [17]. and Canada [18]. In Greece, during 1996–2006 brucellosis, echinococcosis, salmonellosis, and toxoplasmosis were the most common causes of foodborne illnesses, being responsible for 70% of the DALY [19]. The OzFoodNet network was established by the Australian Government Department of Health in 2000 to collaborate nationally to investigate foodborne disease [20, 21]. Under this program, epidemiologists investigate outbreaks of enteric infection conduct studies on the burden of illness, and coordinate national investigations into foodborne disease outbreaks (FBDOs). The system highlighted the misrepresentation of the true burden of outbreaks of gastroenteritis due to under-reporting. There are two foodborne disease surveillance systems in Japan, one for food poisoning, and the other cover pathogens identification [16]. Since 2011, China has established a web-based foodborne disease surveillance platform for FBDOs, early warning of sudden food safety incidents, and research on foodborne disease burden [23]. This platform includes the Foodborne Disease Outbreaks Surveillance System (FDOSS), the Foodborne Disease Surveillance and Reporting System (FDSRS), the National Molecular Traceability Network for Foodborne Diseases (TraNet), and other surveillance systems.

The main surveillance system in India is the IDSP, a decentralized system that collects data on various diseases, including foodborne disease outbreaks throughout the country. The IDSP uses a syndromic surveillance approach that includes an increase in the number of people reporting diarrhea, vomiting, or other foodborne illness symptoms. The IDSP collects data on these outbreaks through laboratory confirmation. In addition to the IDSP, several other surveillance systems collect data on foodborne pathogens in India. These include, the FSSAI surveillance system, the National Rural Drinking Water Programme (NRDWP) [24]. which ensures safe and potable drinking water for all, under the Swachh Bharat Mission campaign that makes initiatives in maintaining cleanliness and hygiene. However, the IDSP is a resource-intensive system and reports only foodborne disease outbreaks. FSSAI is responsible for ensuring food safety and standards at the production, procurement and consumption levels through the ‘Eat Right India’ movement. Currently, India does not have any systematic investigation on major foodborne pathogens prevailing in each region and their potential to cause foodborne diseases and outbreaks. In a country like India with a huge population, different cultures and food practices, it is difficult to make specific food safety guidelines and policies, without strong evidence and data support.

The ICMR-FoodNet database is a valuable resource for public health officials and researchers in the NER. Significant efforts have been made to support and upscale laboratory services in northeast India. This ICMR endeavor is focused on producing scientific evidence to describe risk management decisions and holds significant national importance. ICMR-FoodNet plays a vital role in strengthening India's food control systems with the strategic priorities delineated in the WHO Global Strategy for Food Safety 2022-2030 roadmap [4]. Furthermore, this initiative actively addresses the three global indicators established by WHO [4]. to assess the efficacy and appropriateness of national food safety systems. It involves the collection of accurate data on the incidence of diarrheal diseases and outbreaks originating from contaminated food consumption through an integrated sentinel surveillance of foodborne disease pathogens. This strategy will establish the foundation for fostering multisectoral collaboration with key stakeholders for food safety reforms and policy, promoting a unified approach to food control.

The recommendations and scientific evidence generated from this study would strengthen national food safety and management, ensuring conformity with global standards. Although distinct studies were conducted in India previously, this is the first systematic study, with a uniform methodology, focused on major pathogens. ICMR-FoodNet gives reliable data to compare with global estimates of foodborne illnesses and contributes to the formulation of plans for responding to food safety incidents and emergencies, ensuring effective preparedness and response to emergencies related to foodborne disease. Moreover, the project aims to create a surveillance and research platform that can be customized and employed in various regions across India. Additionally, it can be adopted by other developing countries, thereby contributing to the generation of global data on foodborne pathogen surveillance.

The burden of foodborne diseases in India is huge, and hence early identification, and monitoring through a strong surveillance system for identification of trends, risk factors, and disease burden is the need of the hour. Also, in the era of AMR, it is important to understand the transmission of genes encoding antimicrobial resistance, by tracing the food chain through the one health approach and multidisciplinary action to comprehend the AMR transmission. ICMR initiated this project to reduce the incidence of foodborne diseases and outbreaks in northeast India. Also, to strengthen the foodborne pathogen survey and research network in the country. Arunachal Pradesh, Assam, Sikkim, and Tripura were the first four northeastern states in India to establish the ICMR-FoodNet program, followed by Mizoram Manipur, Meghalaya, and Nagaland, which will serve as the cornerstone for future country-wide scale-up of this surveillance program. A preliminary analysis of data collected using more than 13,000 food/environmental samples and more than 3000 clinical samples indicated that the prevalence of several enteric pathogens. Thirteen foodborne disease outbreaks have been identified in this study. About 200 bacterial strains were identified and stored in the microbial repository. We are in the process of in-depth data analysis including extensive genetic characterization of bacteria strains.

The program's contribution to the nation encompasses several key aspects (1) *Enhancing State-Level Laboratories*: capacity enhancement of the state-level laboratories to be able to function as state-of-the-art facilities for isolating and identifying foodborne pathogens, and serve as reference centers for foodborne outbreak investigations. Real-time data generated by these centers during public health emergencies, such as foodborne disease outbreaks, will help the state governments and policymakers in implementing evidence-based interventions promptly. (2) *Government Partnerships*: establishing partnerships with government organizations like IDSP, FSSAI, and State Health Authority will address a neglected public health issue that is currently a global concern. (3) *OH Approach to AMR Gene Transmission*: The widespread utilization of antimicrobials in farming and agriculture has led to the swift rise of AMR pathogens. Adopting an OH approach to trace the transmission of AMR genes will help in identifying the emerging trend of AMR from the foods/environment [24]. (4) *Data Generation for Policy Development*: data generation on foodborne pathogens and their AMR, will support the development of food safety policies. (5) *Road map to guide development of Global Surveillance Networks:* provide guidance and a structural framework for other developing countries to establish surveillance networks, in line with the WHO recommendations.

The ICMR-FoodNet program has already recognized the significance of public awareness and has integrated a dedicated awareness initiative into its framework. Specifically, we have been actively conducting school health and community awareness programs across the four states involved in the initiative. Furthermore, we are currently in the process of developing a comprehensive education campaign tailored to the specific needs of the target audience. This campaign, entitled "Analysis of knowledge, perception, and practice of food hygiene among school-going children, food handlers, and community leaders: A study in eight states of North East India," is accompanied by a detailed questionnaire. The selection of study sites has been informed by outbreak data collected from state health authorities, ensuring a targeted and effective approach in educating communities about foodborne diseases and preventive measures.

## Data Availability

All data underlying the results are available as part of the article and no additional source data are required.
